# The Probiotic Identity Card: A Novel “Probiogenomics” Approach to Investigate Probiotic Supplements

**DOI:** 10.3389/fmicb.2021.790881

**Published:** 2022-01-21

**Authors:** Gabriele Andrea Lugli, Giulia Longhi, Giulia Alessandri, Leonardo Mancabelli, Chiara Tarracchini, Federico Fontana, Francesca Turroni, Christian Milani, Francesco Di Pierro, Douwe van Sinderen, Marco Ventura

**Affiliations:** ^1^Laboratory of Probiogenomics, Department of Chemistry, Life Sciences, and Environmental Sustainability, University of Parma, Parma, Italy; ^2^GenProbio Srl, Parma, Italy; ^3^Microbiome Research Hub, University of Parma, Parma, Italy; ^4^Velleja Research, Milan, Italy; ^5^Digestive Endoscopy Unit and Gastroenterology, Fondazione Poliambulanza, Brescia, Italy; ^6^APC Microbiome Institute and School of Microbiology, Bioscience Institute, National University of Ireland, Cork, Ireland

**Keywords:** genomics, metagenomics, probiotics, cell viability, flow cytometry

## Abstract

Probiotic bacteria are widely administered as dietary supplements and incorporated as active ingredients in a variety of functional foods due to their purported health-promoting features. Currently available probiotic products may have issues with regards to their formulation, such as insufficient levels of viable probiotic bacteria, complete lack of probiotic strains that are stated to be present in the product, and the presence of microbial contaminants. To avoid the distribution of such unsuitable or misleading products, we propose here a novel approach named Probiotic Identity Card (PIC), involving a combination of shotgun metagenomic sequencing and bacterial cell enumeration by flow cytometry. PIC was tested on 12 commercial probiotic supplements revealing several inconsistencies in the formulation of five such products based on their stated microbial composition and viability.

## Introduction

In 2009 a novel discipline called probiogenomics was coined to provide insights into the diversity of probiotic bacteria aimed at revealing the molecular basis for their health-promoting activities (Ventura et al., 2009). In this context, the availability of probiotic genome sequences significantly expanded our understanding of the biology of these microorganisms (Ventura et al., 2012). Nonetheless, classical microbiological techniques are currently considered the gold standard for probiotic identification, classification, and enumeration (Chiron et al., 2018). However, these techniques are time-consuming and not always accurate when it comes to bacterial identification. In fact, most culture-based methods can only discriminate bacteria at the genus level and only detect microorganisms that can be cultivated (Chiron et al., 2018). In this context, several studies have described efforts to identify the microbial composition of commercial probiotic products that are sold to the US and European markets, encountering products lacking viable bacteria and/or with microbial compositions that deviate from the composition declared by the producers (Drago et al., 2010; Toscano et al., 2013). In recent years, next-generation sequencing technologies have enabled accurate evaluation of the relative abundance of (probiotic) microbes in a sample by targeting the 16S rRNA-encoding gene, thereby avoiding culture-dependent approaches (Morovic et al., 2016; Patro et al., 2016).

More recently, metagenomic sequencing has allowed compositional analysis of 10 probiotic supplements through 16S rRNA gene-associated sequencing and Whole Metagenome Shotgun (WMS) sequencing (Lugli et al., 2019). This analysis revealed inconsistencies of the bacterial presence in four out of 10 probiotic formulations assayed. Nonetheless, using the latter approach, enumeration of probiotic cells in each probiotic supplement was still missing, making it impossible to evaluate the viable count as previously identified by culture-based methods. Flow cytometry (FC) is used extensively in the field of microbiology to count bacteria and determine their viability and metabolic activities (Mudroňová, 2015; Chiron et al., 2018). Furthermore, it has recently been shown that FC is a valid analytical method to quantify lactic acid bacteria (Pane et al., 2018).

We here describe a novel analytic approach, named Probiotic Identity Card (PIC), which was initiated to improve the previously proposed Genetic Identity Card protocol (Lugli et al., 2019) through the use of FC assays to determine absolute abundance and viability of probiotic microorganisms in a given product/sample. In addition, gene-targeted metagenomic analyses involving the 16S rRNA gene and ITS profiling have been replaced by shotgun metagenomics, allowing probiotic classification at species level using a single sequencing methodology.

## Results and Discussion

### The Probiotic Identity Card Workflow

**FIGURE 1:**
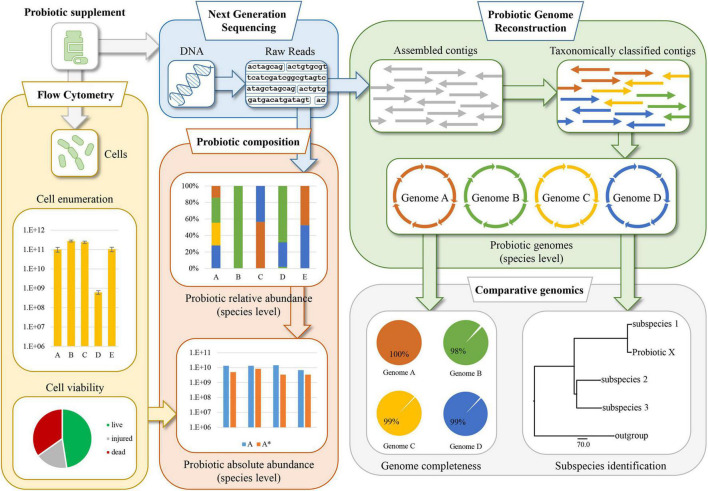
Schematic representation of the probiogenomics-based approach named Probiotic Identity Card (PIC). The methodology involves a whole metagenome shotgun (WMS) analysis followed by taxonomic classification of the reads and genomic reconstruction of the probiotic chromosomes. Then, two flow cytometry assays allow cell enumeration and viability, generating data which together with the WMS analysis are used to determine the integrity and quality of the probiotic supplement formulation.

To perform a detailed microbial compositional assessment of probiotics, probiogenomics approaches were implemented involving next-generation sequencing and multiple FC assays. The workflow of this approach, schematically illustrated in Figure 1, consists of an initial step in which powder-based probiotic supplements are subjected to WMS sequencing. Sequenced DNA was then taxonomically classified at species level to reveal the relative abundance of each microorganism identified in the sample. At the same time, FC assays were performed using serial dilutions of the probiotic supplements, in order to enumerate bacterial cells and reveal their viability using dyes capable to distinguish live cells from dead cells based on cell membrane integrity. Following this, normalization of the WMS sequencing results was performed to estimate the absolute abundance of each viable probiotic strain within each sample assayed. These analyses were complemented by the enumeration of probiotic cells, and the evaluation of their viability. Another important step of the PIC protocol includes the probiotic genome sequence reconstruction based on WMS data obtained from the sequencing methodology, and the completeness of the assembled chromosomes was further validated using *in silico* programs aimed at identifying marker genes. Finally, where the probiotic formulation included microorganisms taxonomically classified as subspecies, the accuracy of the taxonomic identification was verified using a pangenome-based approach. In this context, a phylogenetic tree was built using multiple type strain sequences of the correlated subspecies.

### Taxonomical Dissection of Probiotic Supplements

**FIGURE 2:**
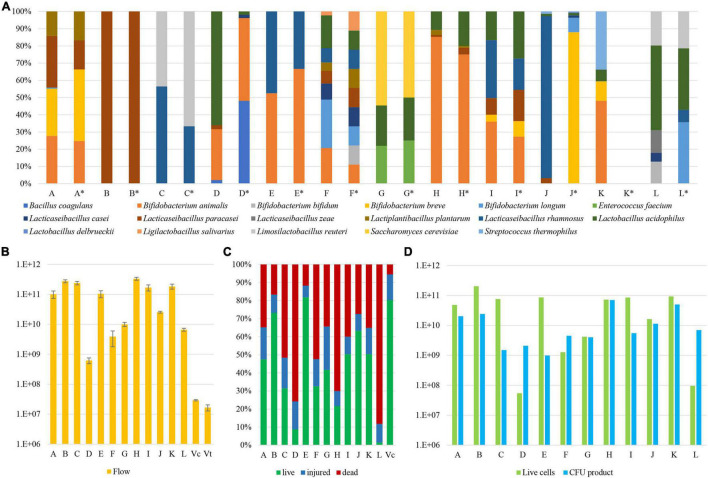
Microbial composition, quantification, and viability of probiotic supplements. Panel **(A)** displays the relative abundance of each microbial species identified in the analyzed probiotic samples. Each probiotic supplement is reported with an identification letter from A to L, which declared composition is listed in detail in Table 1 and reported in pillars marked with an asterisk. Panel **(B)** shows the quantification of the absolute number of microbial cells within each probiotic product. Pillars Vc and Vt report the cell enumeration data of a control sample by means of a flow cytometry assay and a counting chamber, respectively. Panel **(C)** depicts the percentage of viable, injured, and dead cells among individual samples. Finally, panel **(D)** exhibits the normalized number of viable cells (green) next to the value as stated by the producers (blue).

Twelve powder-based probiotic supplements were selected and named A to L to retain anonymity of the products and their commercial origin (Table 1). WMS sequencing was performed to check the microbial composition stated on the packaging of the product. Sequencing outputs ranged from 0.5 to 5.7 million paired-end reads per sample (Supplementary Table 1), allowing an accurate assessment of the probiotic species included in each supplement. In detail, the disparity of sequenced reads obtained from samples was directly proportional to the number of putative different probiotic strains harbored by each probiotic supplement (Table 1). Accordingly, the taxonomic classification of the total amount of 35 million reads allowed the identification of four species of *Bifidobacterium*, i.e., *Bifidobacterium animalis*, *Bifidobacterium bifidum*, *Bifidobacterium breve*, and *Bifidobacterium longum*, and eight species of *Lactobacillus*, i.e., *Lactobacillus acidophilus*, *Lactobacillus casei* (recently reclassified as *Lacticaseibacillus casei*), *Lactobacillus paracasei* (recently reclassified as *Lacticaseibacillus paracasei*), *Lactobacillus plantarum* (recently reclassified as *Lactiplantibacillus plantarum*), *Lactobacillus reuteri* (recently reclassified as *Limosilactobacillus reuteri*), *Lactobacillus rhamnosus* (recently reclassified as *Lacticaseibacillus rhamnosus*), *Lactobacillus salivarius* (recently reclassified as *Ligilactobacillus salivarius*), and *Lactobacillus zeae* (recently reclassified as *Lacticaseibacillus zeae*) (Figure 2; Zheng et al., 2020). Furthermore, *Bacillus coagulans*, *Enterococcus faecium*, *Saccharomyces cerevisiae*, and *Streptococcus thermophilus* species were also detected (Figure 2).

**TABLE 1:** Probiotic data reported on the products.

Code^#^	Probiotic species	N° species	CFU (∼10^9^)^#^
A	*Bifidobacterium animalis* subsp. *lactis*	4	20
	*Bifidobacterium breve*		
	*Lactobacillus paracasei (Lacticaseibacillus paracasei)*		
	*Lactobacillus plantarum (Lactiplantibacillus plantarum)*		

B	*Lactobacillus casei (Lacticaseibacillus casei)*	1	24

C	*Lactobacillus reuteri (Limosilactobacillus reuteri)*	2	1.5
	*Lactobacillus rhamnosus (Lacticaseibacillus rhamnosus)*		

D	*Bacillus coagulans*	4	2
	*Bifidobacterium animalis* subsp. *lactis*		
	*Lactobacillus acidophilus*		
	*Lactobacillus casei (Lacticaseibacillus casei)*		

E	*Bifidobacterium animalis* subsp. *lactis*	9	1
	*Bifidobacterium bifidum*		
	*Bifidobacterium longum* subsp. *longum*		
	*Lactobacillus acidophilus*		
	*Lactobacillus casei (Lacticaseibacillus casei)*		
	*Lactobacillus paracasei (Lacticaseibacillus paracasei)*		
	*Lactobacillus plantarum (Lactiplantibacillus plantarum)*		
	*Lactobacillus rhamnosus (Lacticaseibacillus rhamnosus)*		
	*Lactobacillus salivarius (Ligilactobacillus salivarius)*		

F	*Bifidobacterium animalis* subsp. *lactis*	2	4.5
	*Lactobacillus rhamnosus (Lacticaseibacillus rhamnosus)*		

G	*Enterococcus faecium*	3	4
	*Lactobacillus acidophilus*		
	*Saccharomyces cerevisiae*		

H	*Bifidobacterium animalis* subsp. *lactis*	4	70
	*Lactobacillus acidophilus*		
	*Lactobacillus paracasei (Lacticaseibacillus paracasei)*		
	*Lactobacillus plantarum (Lactiplantibacillus plantarum)*		

I	*Bifidobacterium animalis* subsp. *lactis*	5	5.5
	*Bifidobacterium breve*		
	*Lactobacillus acidophilus*		
	*Lactobacillus paracasei (Lacticaseibacillus paracasei)*		
	*Lactobacillus rhamnosus (Lacticaseibacillus rhamnosus)*		

J	*Bifidobacterium breve*	7	11
	*Bifidobacterium longum* subsp. *infantis*		
	*Lactobacillus acidophilus*		
	*Lactobacillus casei (Lacticaseibacillus casei)*		
	*Lactobacillus delbrueckii* subsp. *bulgaricus*		
	*Lactobacillus rhamnosus (Lacticaseibacillus rhamnosus)*		
	*Streptococcus thermophilus*		

K	*Bifidobacterium animalis* subsp. *lactis*	4	50
	*Bifidobacterium breve*		
	*Lactobacillus acidophilus*		
	*Streptococcus thermophilus*		

L	*Bifidobacterium longum* subsp. *infantis*	4	7
	*Lactobacillus acidophilus*		
	*Lactobacillus reuteri (Limosilactobacillus reuteri)*		
	*Lactobacillus rhamnosus (Lacticaseibacillus rhamnosus)*		

*^#^Probiotic names and CFU of each strain are not reported to keep anonymity of probiotic supplements.*

The analysis showed that probiotic products C, E, G, H, I, and K accurately reflect the bacterial composition as declared by the producer. In contrast, three probiotic supplements revealed that certain species were not present in the product, i.e., *B. bifidum* (product F), *B. breve*, *B. longum*, and *Lactobacillus delbrueckii* (product J), and *B. longum* and *L. rhamnosus* (product L) (Figure 2). Furthermore, a more common inconsistency among the analyzed probiotic supplements was the presence of the *L. paracasei* taxon in samples B, D, and J, instead of the declared *L. casei* species (Table 1). This is a well-known issue since strains belonging to *L. casei* and *L. paracasei* are phenotypically and genotypically closely related (Huang et al., 2018). Furthermore, analysis of two probiotic supplements revealed the presence of additional bacteria not declared by the producers, i.e., bacteria belonging to *B. longum* (product A), and *B. bifidum*, *L. casei*, and *L. zeae* (product L) (Figure 2).

Altogether, through WMS sequencing and subsequent taxonomic profiling of the sequences, we were able to precisely depict the microbial composition of the assessed samples (Figure 2). If ignoring the *L. casei*-*paracasei* misclassification, probiotic supplements A and F revealed a minor contamination of *B. longum* (0.7%) and the lack of a declared *B. bifidum* strain, respectively, while the observed composition of probiotic supplements J and L was shown to be inconsistent with their stated formulations. In particular, probiotic supplement L appeared to contain three unexpected microorganisms, of which *L. zeae* does not even belong to a generally accepted probiotic species.

### Quantification and Viability of Probiotic Strains

Even though WMS sequencing coupled with a bioinformatics approach does allow for a quick and accurate assessment of the microbial composition of probiotic supplements, the application of this approach can only unveil the relative abundance of microorganisms in each sample. Therefore, a flow cytometry (FC) assay was employed to enumerate the actual microbial cell number in each sample, thereby providing information on the absolute number of bacterial cells in the probiotic supplement. Based on the associated information leaflets of the analyzed probiotic supplements, the predicted number of colony-forming units (CFU) ranged from one billion to 70 billion per capsule (Table 1). However, our FC analyses highlighted that bacterial cell numbers of these probiotic supplements ranged from 6.20 × 10^8^ (stdev 1.38 × 10^8^) in sample D to 3.34 × 10^11^ (stdev 3.79 × 10^10^) in sample H (Supplementary Table 2), highlighting that samples D and F contain cell numbers that are already lower than the viable CFU declared by the producer (Figure 2). To validate the accuracy of this approach, an FC assay was further performed on a mock community encompassing two strains belonging to *B. bifidum* and *L. rhamnosus* species with a bacterial load of 1.67 × 10^7^ CFU (stdev 3.82 × 10^6^). Thus, cell enumeration by FC resulted in a number equaling 2.93 × 10^7^ CFU (stdev 1.55 × 10^6^), confirming the reliability of the FC assay established in this study (>0.05 Mann-Whitney *U* test) (Figure 2).

A crucial feature of probiotic microorganisms is represented by their ability to interact with the human gut through the production of different metabolites (Lahtinen, 2012; Sánchez et al., 2017). Based on scientific literature published on this topic, health benefits conferred by viable probiotics are considered to be more prominent than those achieved by non-viable probiotics, also known as postbiotics (Żółkiewicz et al., 2020). Thus, the viability of microorganisms from each probiotic was also investigated by means of FC using dyes that distinguish live cells from dead cells based on cell membrane integrity. The percentage of viable cells with respect to the total load of bacterial cells as determined by FC ranged from 1.5% in probiotic L to 81.9% in probiotic E, with an average of 42% of viable cells among probiotic supplements (Figure 2). As a result of the analysis, products C, D, F, H, and L, revealed that the proportion of viable cells was <40%, indicative of serious issues concerning the efficacy of these probiotic products. In addition, similar as described above for cell enumeration, an FC assay was performed on a mock community encompassing viable *B. bifidum* and *L. rhamnosus* taxa collected at the end of their exponential growth phase demonstrating the estimated presence of 5.5% non-viable cells, thus validating the approach (Figure 2).

**FIGURE 3:**
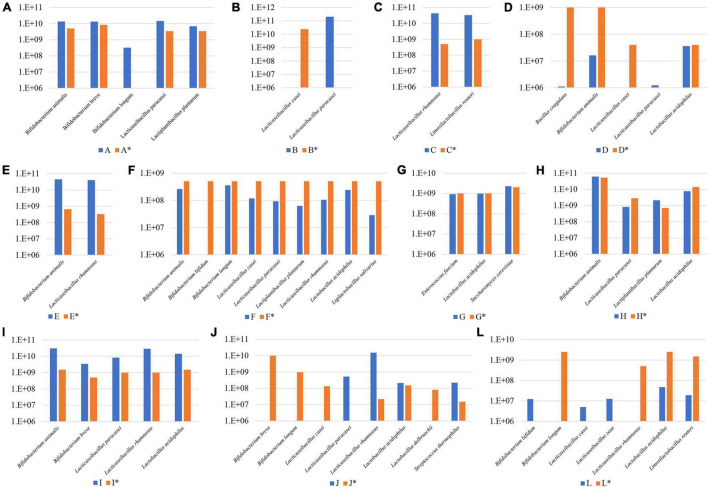
Absolute abundance values for microbes in a variety of probiotic supplements. Panel **(A–L)** exhibit the absolute abundance of each probiotic species among 11 supplements reported with an identification letter from A to L. Declared viability values of each microbial species is reported in pillars marked with an asterisk.

Subsequently, FC data obtained for each probiotic supplement was further employed to normalize the total reads determined by WMS experiments according to a previously described method (Lugli et al., 2020), thereby allowing us to estimate the absolute abundance of each probiotic strain of the probiotic supplements assayed (Figure 3). In detail, the composition of probiotic supplements C, E, G, H, and I were shown to be in near perfect agreement with what was declared by the producers for both the presence and absolute load, except for *L. paracasei* in sample H that exhibited an estimated cell number of 8.27 × 10^8^ instead of 2.8 × 10^9^ (Figure 3). Furthermore, the microbial composition of probiotic A was also in very good correspondence with that stated in the accompanying information, except for apparent contamination of *B. longum* (present at 3.24 × 10^8^), which represents a relatively minor fraction when compared to the total estimated number of viable cells of 4.83 × 10^10^. Since the information accompanying supplement K does not report the CFU of each individual strain (while the combined CFU of the product is reported in Supplementary Table 2), we did not include its absolute composition in this discussion. In contrast, probiotic supplements B, D, F, J, and L revealed serious discrepancies with respect to the absolute microbial content as stated by the manufacturers of these products (Figure 3). In this context, we observed probiotic supplementations that were shown to contain much lower viable cells when compared to the number stated by the producers, in particular samples D (5.44 × 10^7^ vs. 2.08 × 10^9^) and L (9.62 × 10^7^ vs. 7 × 10^9^). Furthermore, we also noted formulations in which a single strain is numerically far more dominant compared to other strains in that same supplement as observed in sample J by *L. rhamnosus* (1.52 × 10^10^ vs. 2.2 × 10^7^) (Figure 3).

### Genome Reconstruction of Probiotic Strains

**FIGURE 4:**
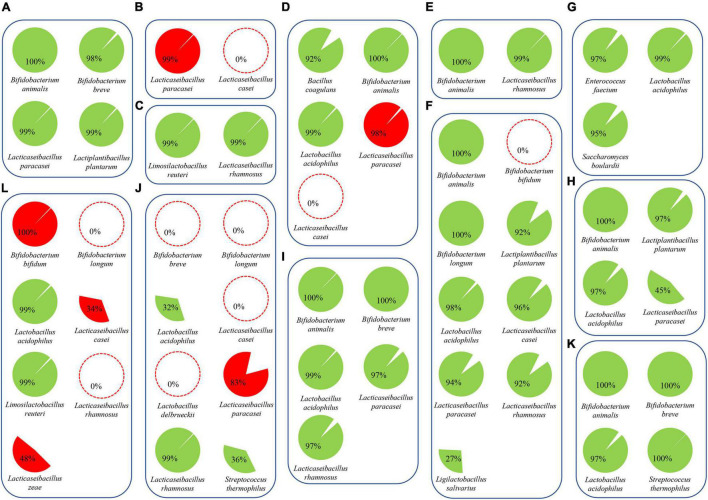
Assembled genomes from probiotic supplements. Reconstructed microbial genomes are represented by cake diagrams arranged in boxes for each of the 12 probiotic products (**A–L**; see Table 1). Missing microbes are reported as red dotted circles, while bacterial genomes not declared by the producers are highlighted in red. The percentage of each cake diagram corresponds to the completeness of the reconstructed chromosomes.

The high number of sequenced paired-end reads obtained for each probiotic product allowed us to perform metagenomic assembly to reconstruct the genome sequence of each probiotic microorganism. Assembled chromosomal sequences were taxonomically classified at the species level using a set of databases of validated reference genomes (Milani et al., 2021). Accordingly, shotgun sequencing data allowed the genome reconstruction of 46 chromosomal sequences encompassing strains belonging to *Bacillus coagulans*, *B. animalis*, *B. bifidum*, *B. breve*, *B. longum*, *E. faecium*, *L. acidophilus*, *L. casei*, *L. paracasei*, *L. plantarum*, *L. reuteri*, *L. rhamnosus*, *L. salivarius*, *L. zeae*, *Saccharomyces cerevisiae*, and *Streptococcus thermophilus* (Figure 4). Likewise, reconstruction of such genome sequences revealed a microbial strain distribution across the 12 probiotic supplements identical to that predicted in the taxonomic classification of the short-read sequences (Figure 2). In a similar fashion, assembled reads of product A unveiled a contig of 42 Kb classified as *B. longum*, confirming the presence of putative contamination of this species in the formulation. Collected data further validated that products B, D, and F displayed some minor issues in the formulation represented by the misclassification of *L. paracasei* in *L. casei* and the absence of *B. bifidum* in sample F (Figure 4). In contrast, products J and L lacked multiple strains and showed high contamination of other bacterial cells verifying the presence of *L. zeae* in sample L (Figure 4). The (lack of) completeness of each reconstructed bacterial chromosome highlighted those strains previously estimated in low abundance within samples (Figure 2), resulting in the partial genomic reconstruction of *L. acidophilus* (32% in sample J), *L. paracasei* (45% in sample H), *L. salivarius* (27% in sample F), and *Streptococcus thermophilus* (36% in sample J) (Figure 4 and Supplementary Table 3).

Since profiling of shotgun metagenomics data at the subspecies level is still very challenging, pangenome-based classification using genome sequences of related type strains have been used. In this context, 9 out of the 12 assessed probiotic supplements were shown to contain bacteria belonging to *B. animalis* subsp. *lactis*, *B. longum* subsp. *infantis*, and *B. longum* subsp. *longum*. This analysis in the PIC workflow allowed us to build a pangenome-based phylogenetic tree to classify each strain at subspecies level (Supplementary Figure 1). Results highlighted that reconstructed *B. animalis* genomes in samples A, D, E, F, H, I, and K are highly related from a phylogenetic perspective, all belonging to the subspecies *B. animalis* subsp. *lactis*, while the reconstructed *B. longum* in sample F was shown to belong to the subspecies *B. longum* subsp. *longum*. Thus, using reference genomes, we were able to verify all probiotic strains classified as subspecies.

## Conclusion

Using a combination of WMS sequencing and FC analyses, we characterized the microbial contents of commercial, powder-based, probiotic products, unveiling their presence as well as abundance and viability. The PIC pipeline described in this work improves the previously proposed Genetic Identity Card (Lugli et al., 2019), removing redundant sequencing experiments, such as 16S rRNA gene and ITS profiling, and allowing the precise enumeration of viable cells of each probiotic strain present in the probiotic supplement. Thus, the PIC approach can validate the probiotic formulation in terms of presence and absolute abundance of each probiotic microorganism, estimating the stated accuracy of the final product. Furthermore, no additional culturomic-based experiments are required, removing bias related to the differential grow capability of strains on different substrates. The PIC approach is also particularly suitable for dissecting the composition and verifying cell viability of probiotic supplements that encompass multiple probiotic strains (also known as mixes of probiotic bacteria) that are otherwise difficult to assess using standard culture-based approaches.

Using the PIC approach, five probiotic supplements out of 12, i.e., >40%, reveal inconsistencies in the formulations regarding what was declared, thus raising concerns about the current protocols applied by the internal quality checks of the probiotic supplement producer. Hopefully, molecular approaches such as the one described in this study will be established in the future by national agencies charged with quality control of probiotic products on the market.

## Materials and Methods

### Probiotic Products Selection

Twelve powder-based probiotic supplements were randomly selected from the Italian market to analyze probiotic products composed of single- and multi-strain microorganisms. Commercial names of collected products retrieved from pharmacies were renamed to observe anonymity of the producers. In detail, probiotic supplements were named with alphabetic letters, from A to L, and their microbial composition declared by the anonymous producers was listed in Table 1.

### Microbial DNA Extraction

Probiotic supplements were dissolved and homogenized thoroughly in Phosphate Buffer Solution (PBS; pH 6.5) to obtain the primary 1:10 dilution of each tested sample. Subsequently, 1 mL of each resuspended freeze-dried sample was subjected to chromosomal DNA extraction using the ZymoBIOMICS DNA Miniprep Kit (Zymo Research, D4300) following the manufacturer’s instructions. Then, each probiotic supplement’s DNA concentration and purity were investigated employing a Picodrop microtiter Spectrophotometer (Picodrop, Hinxton, United Kingdom).

### Shotgun Metagenomics Sequencing

According to the manufacturer’s instructions, DNA library preparation was performed using the Nextera XT DNA sample preparation kit (Illumina, San Diego, CA, United States). First, 1 ng input DNA from each probiotic supplement was used for the library preparation which underwent fragmentation, adapter ligation, and amplification. Then, Illumina libraries were pooled equimolarly, denatured, and diluted to a concentration of 1.5 pM. Next, DNA sequencing was performed on a NextSeq 550 instrument (Illumina) using a 2X 150 bp Output sequencing Kit together with a deliberate spike-in of 1% PhiX control library.

### Taxonomic Classification of Short Sequenced Reads

Sequenced paired-end reads of each probiotic supplement were subjected to a filtering step removing low-quality reads (minimum mean quality score 20, window size 5, quality threshold 25, and minimum length 100) using the fastq-mcf script^1^ to analyze high-quality sequenced data only. Then, an additional filtering step was performed to remove possible contaminating human DNA sequences from each sample through reads mapping employing the BWA aligner (Li and Durbin, 2009). Filtered reads were then collected and taxonomically classified through the METAnnotatorX2 pipeline (Milani et al., 2021), using a set of databases of reference genomes whose taxonomy was previously validated to maximize the accuracy of homology-based taxonomic classification of reads (Milani et al., 2021).

### Genome Reconstruction of Probiotics Through Whole Metagenome Shotgun Sequencing

Filtered paired-end reads were subjected to whole metagenome assembly using Spades v3.15 (Prjibelski et al., 2020) with default parameters and the metagenomic flag option (–meta) together with k-mer sizes of 21, 33, 55, and 77. Reconstructed chromosomal contig sequences of probiotics were taxonomically classified against manually curated genome databases as reported above for the taxonomic classification of short sequenced reads (Milani et al., 2021). Overall, the METAnnotatorX2 pipeline was used to manage WMS data from read-filtering to taxonomic classification of the assembled contigs (Milani et al., 2018, 2021).

### Flow Cytometry Analyses

From the initial 1:10 dilution of each probiotic supplement, five subsequent 10-fold serial dilutions were prepared in PBS. Then, one mL of bacterial cell dilution was stained with 1 μl mL^–1^ SYBR Green I diluted 1:100 in DMSO (starting from a 10,000X in DMSO; Molecular Probes, Eugene, OR, United States) and incubated in the dark for 15 min before measurement. Count experiments were performed using an Attune NxT flow cytometer (Thermo Fisher Scientific, Waltham, MA, United States) equipped with a blue laser set at 50 mW and tuned to an excitation wavelength of 488 nm. Multiparametric analyses were performed on scattering signals, i.e., forward scatter (FSC) and side scatter (SSC), and SYBR Green I fluorescence was detected on the FL1 channel. Described analysis was performed in triplicate for each probiotic product. Cell debris was excluded from the acquisition analysis by a sample-specific FL1 threshold, and collected data were statistically analyzed with Attune NxT flow cytometer software. The precision of the enumeration method was determined on a mock community encompassing two strains belonging to *B. bifidum* and *L. rhamnosus* species, also coupled with a Thoma counting chamber calculation.

### Microbial Viability Count

Two aliquots of 1 ml of bacterial cell dilution were harvested by centrifugation at 3,000 × *g* for 8 min. Cells were washed twice and resuspended in PBS. One of two aliquots of bacterial suspension was exposed to 70% isopropyl alcohol for 1 h to permeabilize cell membranes and cause cell death. Flow cytometry cell viability assay was carried out on both aliquots using the fluorescent stains SYTO9 (3.34 mM) and PI (20 mM) of LIVE/DEAD BacLight Bacterial Viability kit (ThermoFisher Scientific, Waltham, MA, United States), following the manufacturer’s protocol. Specifically, 1.5 μl of a dye was added to the sample for the single staining assay, while for the double staining assay, 1.5 μl of both dyes was used. Immediately following staining, samples were incubated in the dark for 15 min at room temperature. For instrument parameter adjustment, single-colored controls were used, while non-stained cells were used as a background control. Cell viability assay was performed with an Attune NxT flow cytometer (ThermoFisher Scientific, Waltham, MA, United States), and all data were analyzed with Attune NxT flow cytometer software. The precision of the viability count was determined on a mock community encompassing two strains belonging to *B. bifidum* and *L. rhamnosus* species.

### Comparative Genomics

The quality of reconstructed probiotic genomes was estimated for their completeness and contamination using CheckM v1.1.3 (Parks et al., 2015) and BUSCO v5 (Seppey et al., 2019). Then, pangenome calculations for subspecies identification of reconstructed probiotics were performed using the pangenome analysis pipeline PGAP (Zhao et al., 2012). Predicted proteomes were screened for orthologs between groups using BLAST analysis (cutoff E-value of <1 × 10^––5^ and 50% identity across at least 80% of either protein sequence). The resulting output was clustered into protein families through MCL (graph theory-based Markov clustering algorithm) using the gene family method. Using this approach, phylogenetic trees were built, including genetic sequences of type strain retrieved from NCBI. The core genome trees were produced using the software FigTree^2^.

## Data Availability Statement

Shotgun metagenomics data are accessible through SRA study accession number PRJNA767508.

## Author Contributions

GAL performed bioinformatics analyses and wrote the manuscript. GL performed the *in vitro* analyses and edited the manuscript. GA and CT validated the *in vitro* analyses. LM and FF validated the bioinformatics analyses. FT, CM, FD, and DS supervised the project and edited the manuscript. MV supervised the project and designed the study. All authors contributed to the article and approved the submitted version.

## Conflict of Interest

GL and FF were employed by GenProbio Srl. FD was employed by company Velleja Research. The remaining authors declare that the research was conducted in the absence of any commercial or financial relationships that could be construed as a potential conflict of interest.

## Publisher’s Note

All claims expressed in this article are solely those of the authors and do not necessarily represent those of their affiliated organizations, or those of the publisher, the editors and the reviewers. Any product that may be evaluated in this article, or claim that may be made by its manufacturer, is not guaranteed or endorsed by the publisher.

## Footnotes

1
https://github.com/ExpressionAnalysis/ea-utils/blob/wiki/FastqMcf.md

2
http://tree.bio.ed.ac.uk/software/figtree/

## Supplementary Material

Supplementary Figure 1 Pangenome-based phylogenetic trees of probiotic strains. Panel **(A)** shows the pangenome-base phylogenetic tree of *B. animalis* reconstructed genomes, while panel **(B)** exhibits the equivalent tree for *B. longum* probiotic genomes.
