# Supplementary figures and images for: The Probiotic Identity Card: A Novel “Probiogenomics” Approach to Investigate Probiotic Supplements
Source: Front Microbiol. 2022 Jan 21;12:790881. doi: 10.3389/fmicb.2021.790881 (PMC8814603; doi:10.3389/fmicb.2021.790881)

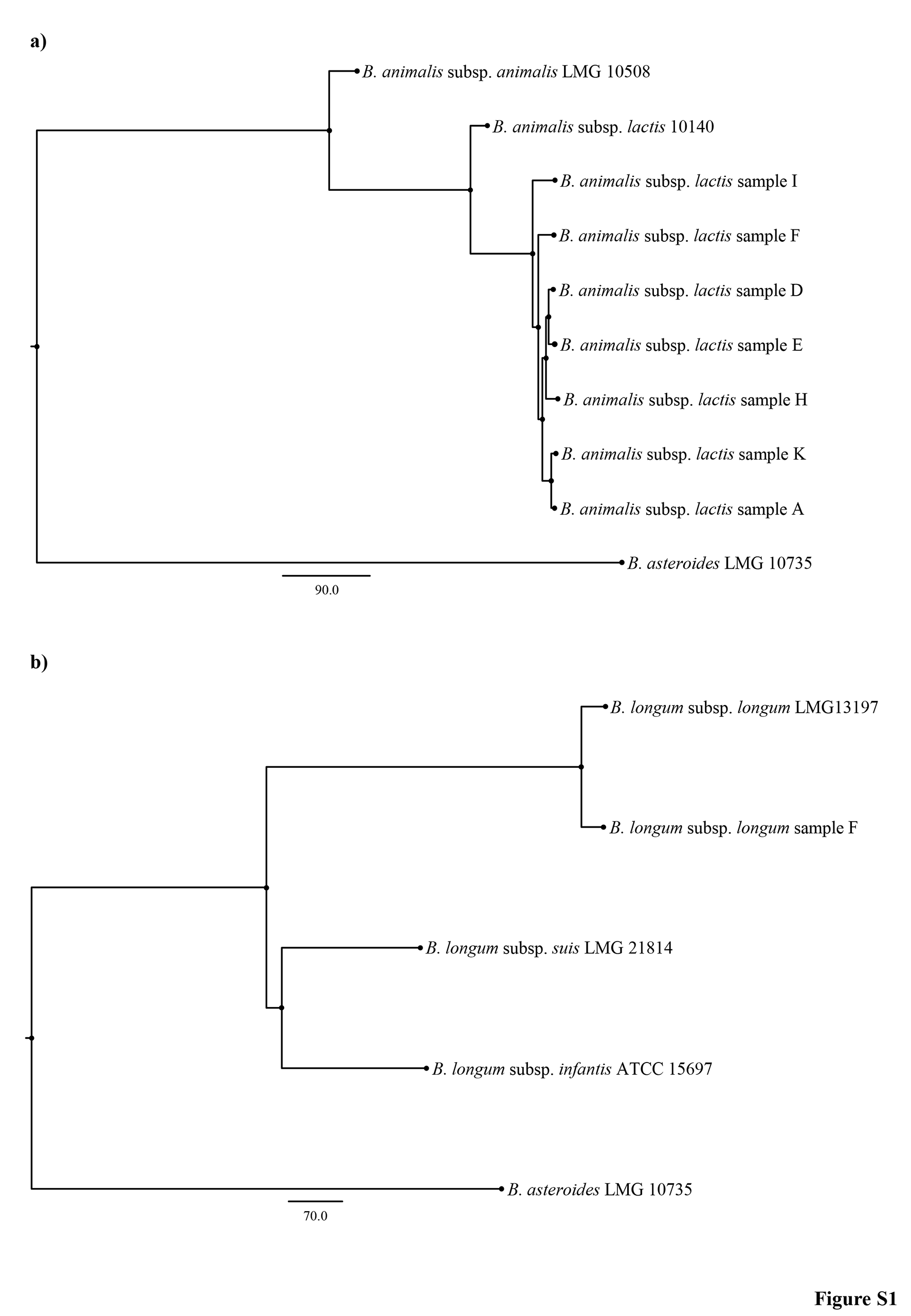

Supplement: Supplementary Figure 1 — Pangenome-based phylogenetic trees of probiotic strains. Panel (A) shows the pangenome-base phylogenetic tree of B. animalis reconstructed genomes, while panel (B) exhibits the equivalent tree for B. longum probiotic genomes. [file Image_1.TIF]
